# Supplementary material for: General and COVID-19-Related Mortality by Pre-Existing Chronic Conditions and Care Setting during 2020 in Emilia-Romagna Region, Italy
Source: Int J Environ Res Public Health. 2021 Dec 15;18(24):13224. doi: 10.3390/ijerph182413224 (PMC8701253; doi:10.3390/ijerph182413224)
Supplement: Supplementary file 1 [file ijerph-18-13224-s001.zip › STATA commands for models.pdf]

* **<u>Poisson models</u>** for **Mortality Rate Ratios (MRR: exp(β1))** estimates:

STATA code:

```
xi: poisson ly x1 i.x2 if sex==i & age_gr==j, e(pop) irr
```

* ly=yearly deaths count (total and for each care setting: hospital, Community / Long-term Hospital, hospice, Long-term Care Facilities, home Care, none of the above) stratified for 5-years age groups and sex

* x1: dummy for year 2020

* x2: 5-years age classes

* age_gr: two age groups 18-74 and 75+ years old

* pop: population at January the 1st of each of the 6 years (offset).

* **<u>Log-binomial models</u>** for **Prevalence Ratios (PR: exp(βd))** estimates:

STATA code:

```
xi: glm y2020 x1 x2 x3 x4 x5 x6 x7 i.x8, link(log) family(binom) eform
```

* y2020: dummy for death occurred in 2020

* x1, …, x7: dichotomic variables, one for each chronic condition (cardiovascular, cancer, respiratory, metabolic, neuropsychiatric, musculoskeletal, none of the above conditions)

* x8: 5-years age classes.
